# Foodborne Illness Acquired in the United States

**DOI:** 10.3201/eid1707.110019

**Published:** 2011-07

**Authors:** Craig W. Hedberg

**Affiliations:** Author affiliation: University of Minnesota, Minneapolis, Minnesota, USA

**Keywords:** burden of illness, foodborne disease surveillance, norovirus, viruses, letter

**To the Editor:** The updated estimates of foodborne illness in the United States reported by Scallan et al. probably overestimate the occurrence of illness caused by unspecified agents because they did not account for the apparent sensitivity of the population survey to the occurrence of norovirus (*1**,**2*). The number of illnesses attributed to unspecified agents was derived from the simultaneous processes of extrapolation and subtraction: extrapolation from the population survey to create a base of diarrheal illnesses and subtraction of known agents from this base. Scallan et al. averaged illness rates from 3 successive population surveys to come up with a rate of 0.6 episodes of acute gastroenteritis per person per year. However, the individual rates were 0.49 (2000–2001), 0.54 (2002–2003), and 0.73 (2006–2007). The 2006–2007 survey was conducted at the time of widespread norovirus activity. The estimated rate of population illness was strongly correlated with the number of confirmed and suspected norovirus outbreaks reported to the Centers for Disease Control and Prevention Foodborne Disease Outbreak Surveillance System during each of the survey periods (300, 371, and 491, respectively; R^2^ = 0.97, p<0.0001). No other known agents were correlated with the population survey rates, and the total numbers of outbreaks were inversely correlated with the population survey data.

The strength of the correlation between norovirus outbreaks and survey results suggests that the population survey is sensitive to norovirus activity and that norovirus may account for much of what is considered to be unspecified. The fact that the highest observed population rate was ≈50% greater than the lowest rate suggests that annual variation in norovirus activity may account for a considerable proportion of what otherwise seems to be unspecified. More thorough and timely investigation and reporting of outbreaks could facilitate the development of models to evaluate the number of illnesses and update them annually.
