# Supplementary material for: Foodborne Illness Acquired in the United States
Source: Emerg Infect Dis. 2011 Jul;17(7):1338–40. doi: 10.3201/eid1707.110019 (PMC3321563; doi:10.3201/eid1707.110019)
Supplement: Related texts

# Reply: Foodborne Illness Acquired in the United States

**Authors:** James R. Johnson

**Affiliations:** Author affiliations: Minneapolis VA Medical Center Minneapolis, Minnesota, USA;

**Keywords:** Keywords: Escherichia coli infections, foodborne illness, antimicrobial resistance, bacteria, extraintestinal, letter

**To the Editor:** The report by Scallan et al. provides a valuable update regarding estimated annual illnesses, hospitalizations, and deaths caused by recognized foodborne pathogens, most of which cause diarrheal disease, in the United States (*1*). However, absent from this study, and from most previous reviews of foodborne illness, was attention to possible extraintestinal disease, especially antimicrobial drug–resistant infections caused by food-source *Escherichia coli* and associated resistance elements.

A growing body of molecular and epidemiologic evidence suggests that a substantial fraction of extraintestinal *E. coli* infections in humans, particularly those involving antimicrobial drug–resistant strains, might be caused by *E. coli* from food animals (*2*). Extraintestinal pathogenic and antimicrobial drug–resistant *E. coli* commonly contaminate retail meat products (*3**,**4*); rates of contamination and resistance associated with “no antibiotics” production methods, labeling, and markets are lower (*4*). In a study of women with acute urinary tract infection, frequent consumption of chicken and pork was associated with isolation of antimicrobial drug–resistant *E. coli* from urine (*5*).

Extraintestinal *E. coli* infections, which include urinary tract infections and sepsis, are more common and result in more hospitalizations and deaths than do infections caused by the classic foodborne pathogens. For example, each year in the United States, an estimated 40,000 deaths are associated with sepsis caused by extraintestinal *E. coli* infection (*6*); only <1,400 deaths are caused by all major classic foodborne pathogens combined (*1*). Therefore, if even a modest fraction (e.g., 5%–10%) of all extraintestinal *E. coli* infections in humans are of foodborne origin—which seems highly plausible, considering the molecular evidence (*2*)—the extent of associated disease may equal or exceed that attributable to the classic foodborne pathogens as estimated by Scallan et al. Greater recognition of this possibility by the public health system is needed so that appropriate attention can be devoted to this neglected, invisible foodborne disease threat.

## References

[1] Scallan E, Hoekstra R, Angulo FJ, Tause RV, Widdowson M-A, Roy SL, Foodborne illness acquired in the United States—major pathogens. Emerg Infect Dis. 2011;17:7–15.2119284810.3201/eid1701.P11101PMC3375761

[2] Johnson JR, Sannes MR, Croy C, Johnston B, Clabots C, Kuskowski MA, Antimicrobial drug–resistant *Escherichia coli* isolates from humans and poultry products, Minnesota and Wisconsin, 2002–2004. Emerg Infect Dis. 2007;13:838–46. 10.3201/eid1306.06157617553221PMC2792839

[3] Johnson JR, McCabe JS, White DG, Johnston B, Kuskowski MA, McDermott P. Molecular analysis of *Escherichia coli* from retail meats (2002–2004) from the United States National Antimicrobial Resistance Monitoring System (NARMS). Clin Infect Dis. 2009;49:195–201. 10.1086/59983019508167

[4] Johnson JR, Kuskowski MA, Smith K, O’Bryan TT, Tatini S. Antimicrobial-resistant and extraintestinal pathogenic *Escherichia coli* in retail foods. J Infect Dis. 2005;191:1040–9. 10.1086/42845115747237

[5] Manges AR, Smith SP, Lau BJ, Nuval CJ, Eisenberg JN, Dietrich PS, Retail meat consumption and the acquisition of antimicrobial resistant *Escherichia coli* causing urinary tract infections: a case–control study. Foodborne Pathog Dis. 2007;4:419–31. 10.1089/fpd.2007.002618041952

[6] Russo TA, Johnson JR. Medical and economic impact of extraintestinal infections due to *Escherichia coli*: focus on an increasingly important endemic problem. Microbes Infect. 2003;5:449–56. 10.1016/S1286-4579(03)00049-212738001

# Reply: Foodborne Illness Acquired in the United States

**DOI:** 10.3201/eid1707.110572

**Authors:** Elaine Scallan, Robert M. Hoekstra, Marc-Alain Widdowson, Aron J. Hall, Patricia M. Griffin

**Affiliations:** Author affiliations: University of Colorado Denver, Aurora, Colorado, USA (E. Scallan);; Centers for Disease Control and Prevention, Atlanta, Georgia, USA (R.M. Hoekstra, M.-A. Widdowson, A.J. Hall, P.M. Griffin)

**Keywords:** *Suggested citation for this article*: Scallan E, Hoekstra RM, Widdowson M-A, Hall AJ, Griffin PM. Foodborne illness acquired in the United States [response]. Emerg Infect Dis [serial on the Internet]. 2011 Jul [*date cited*]. http://dx.doi.org/10.3201/eid1707.110572

**In Response:** We welcome the suggestions by Hedberg (*1*) and Johnson (*2*) on how future estimates of foodborne illness in the United States could be improved (*3**,**4*). We agree with Johnson that our estimates of foodborne illness probably reflect underrecognition of the extent of extraintestinal disease (*2*). Our estimates of foodborne illness caused by major pathogens included 7 pathogens that cause conditions other than gastroenteritis. However, our estimates of foodborne illness caused by unspecified agents relied on reports of acute gastroenteritis. We did not include illness caused by known foodborne agents that do not typically cause gastroenteritis, such as ciguatoxins and some mushroom toxins, for which insufficient data were available to estimate agent-specific episodes of illness (*4*). We agree that urinary tract infections with *Escherichia coli*, most of which come from the patient’s own gut flora and for which the original source may be food, may fall into this category and that the number of such illnesses is large (*5*). Moreover, there are probably unknown or unrecognized agents in the food supply that cause illness other than gastroenteritis. We recognize the need to think of ways to include more of these agents—known and unspecified—in future estimates.

Hedberg noted that rates of acute gastroenteritis in the 3 FoodNet population surveys correlate with the number of reported foodborne norovirus outbreaks and suggested that we may have consequently underestimated illnesses caused by norovirus and overestimated illnesses caused by unspecified agents (*1*). Although such an association between FoodNet survey findings and reported norovirus outbreaks is possible, it should be treated with caution. The annual number of foodborne norovirus outbreaks reported during our study was probably influenced by improvements in diagnosis and surveillance. The steady increase in suspected and confirmed foodborne norovirus outbreaks during 1998–2003 was accompanied by a decrease in foodborne outbreaks of unknown etiology, suggesting that the higher number of norovirus outbreaks reported during 2006–2007 resulted, at least in part, from improved diagnosis. In addition, nonfoodborne outbreaks caused by a new norovirus strain increased during 2002–2003 and were not reflected in the population survey by an increased rate of gastroenteritis (*6*). The surveys varied in how questions were worded and ordered, especially the 2006–2007 survey compared with earlier surveys, and higher rates of acute gastroenteritis might be related to these variations. Moreover, the 2006–2007 increase in gastroenteritis reported by the surveys was driven by increases in diarrhea rather than vomiting (the latter is more suggestive of norovirus illness). The limited number of comparison data points—only 3—and the fact that the data were from different populations (all states vs. 10 FoodNet sites) during slightly different time periods (FoodNet surveys were conducted over 12 months but not in 1 calendar year) also warrant conservative assessment of the association between annual rates of acute gastroenteritis and number of foodborne norovirus outbreaks. Because of uncertainties in both measures, we preferred a rank-based method; the Spearman ρ gives an exact p value of 0.33.

To estimate average annual number of illnesses, we used data from several years (2000–2008) and probability distributions. The fraction of acute gastroenteritis estimated to be attributable to norovirus was wide (6%–26%), so it probably encompassed year-to-year variations in incidence. Not only is norovirus estimated to be the most common known cause of foodborne illness, but it is the illness for which we have the least surveillance information. Better data for norovirus are needed. Studies to examine the association between norovirus activity and reported rates of acute gastroenteritis of unknown cause could be useful. Although the most recent FoodNet survey of gastroenteritis was conducted in 2006, more surveys await funding.

## References

[1] Hedberg CW. Foodborne illness acquired in the United States [letter]. Emerg Infect Dis. 2011;17:1338. 10.3201/eid1707.11001921762617PMC3321563

[2] Johnson JR. Foodborne illness acquired in the United States [letter]. Emerg Infect Dis. 2011;17:1338–9. 10.3201/eid1707.11025621762616

[3] Scallan E, Hoekstra RM, Angulo FJ, Tauxe RV, Widdowson MA, Roy SL, Foodborne illness acquired in the United States—major pathogens. Emerg Infect Dis. 2011;17:7–15.2119284810.3201/eid1701.P11101PMC3375761

[4] Scallan E, Griffin PM, Angulo FJ, Tauxe RV, Hoekstra RM. Foodborne illness acquired in the United States—unspecified agents. Emerg Infect Dis. 2011;17:16–22.2119284910.3201/eid1701.P21101PMC3204615

[5] Manges AR, Smith SP, Lau BJ, Nuval CJ, Eisenberg JN, Dietrich PS, Retail meat consumption and the acquisition of antimicrobial resistant *Escherichia coli* causing urinary tract infections: a case–control study. Foodborne Pathog Dis. 2007;4:419–31. 10.1089/fpd.2007.002618041952

[6] Widdowson MA, Cramer EH, Hadley L, Bresee JS, Beard RS, Bulens SN, Outbreaks of acute gastroenteritis on cruise ships and on land: identification of a predominant circulating strain of norovirus—United States, 2002. J Infect Dis. 2004;190:27–36. 10.1086/42088815195240
